# Supplementary material for: Crosstalk between the Rod Outer Segments and Retinal Pigmented Epithelium in the Generation of Oxidative Stress in an In Vitro Model
Source: Cells. 2023 Aug 30;12(17):2173. doi: 10.3390/cells12172173 (PMC10487269; doi:10.3390/cells12172173)
Supplement: Supplementary file 1 [file cells-12-02173-s001.zip › cells-2501034-supplementary.pdf]

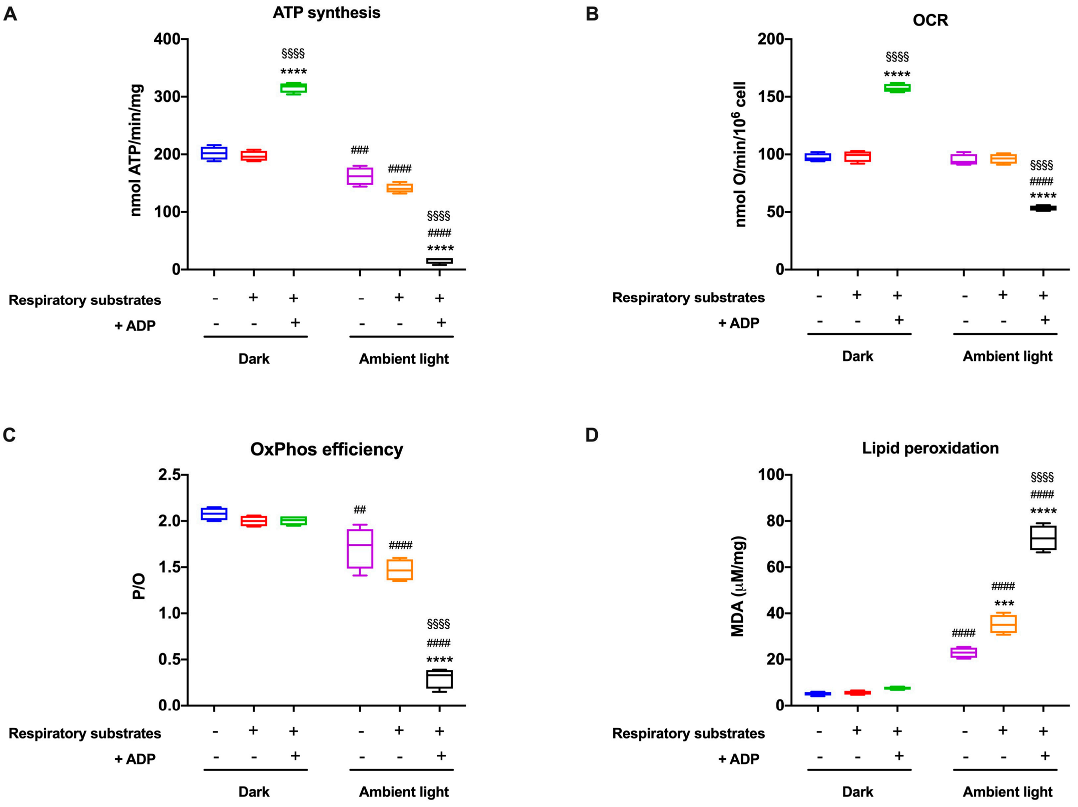

Figure S1. ATP synthesis, oxygen consumption rate, OxPhos efficiency, and lipid peroxidation in rod OSs. All the data reported in this figure are obtained from bovine rod OSs preincubated or not with respiratory substrates (0.6 mM NADH and 20 mM succinate) and/or 0.1 mM ADP and maintained in the dark or exposed to ambient light for 30 min. (**A**) ATP synthesis through F_1F_o-ATP synthase and (**B**) oxygen consumption rate (OCR); in both experiments, 0.1 mM NADH has been added to induce OCR and ATP synthesis. (**C**) P/O value, a marker of OxPhos efficiency, calculated as the ratio between synthetized ATP and consumed oxygen. (**D**) MDA content as a lipid peroxidation marker. Data are representative of four independent replicates (n = 4). *** and **** indicate a significant difference for $p < 0.001$ or 0.0001, respectively, between basal rod OSs and rod OSs incubated with respiratory substrates and/or ADP, both when kept in the dark or exposed to ambient light. ##, ###, and #### indicate a significant difference for $p < 0.01$, 0.001, or 0.0001 between the rod OSs maintained in the dark or exposed to ambient light, under the same respiratory substrates conditions. §§§§ indicates a significant difference for $p < 0.0001$, between the rod OSs treated with only respiratory substrates and those incubated with respiratory substrates + ADP. The data suggest that when rod OSs are maintained in the dark, both in the absence and presence of pretreatment with respiratory substrates or/and ADP, they can produce ATP by consuming oxygen without altering OxPhos efficiency or lipid peroxidation levels. In contrast, when rod OSs are exposed to ambient light for 30 minutes in the presence of respiratory substrates, they show an alteration in aerobic metabolism associated with uncoupling and increased peroxided lipid accumulation. These effects are more evident when rod OSs are incubated with both respiratory substrates and ADP, suggesting that to induce oxidative stress in rod OSs, they must be exposed to light in the presence of all OxPhos-activating substrates.

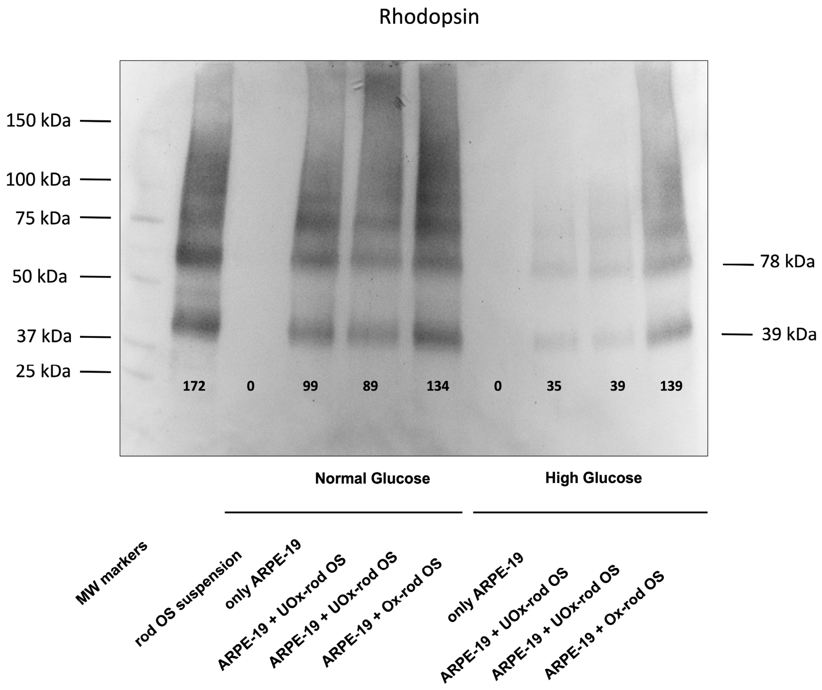

Figure S2. Whole WB signal against rhodopsin quantity in ARPE-19 cell growth media as a marker of cell ability to phagocyte unoxidized or oxidized rod OSs. The WB signal refers to Panel A in Figure 3 of the manuscript.

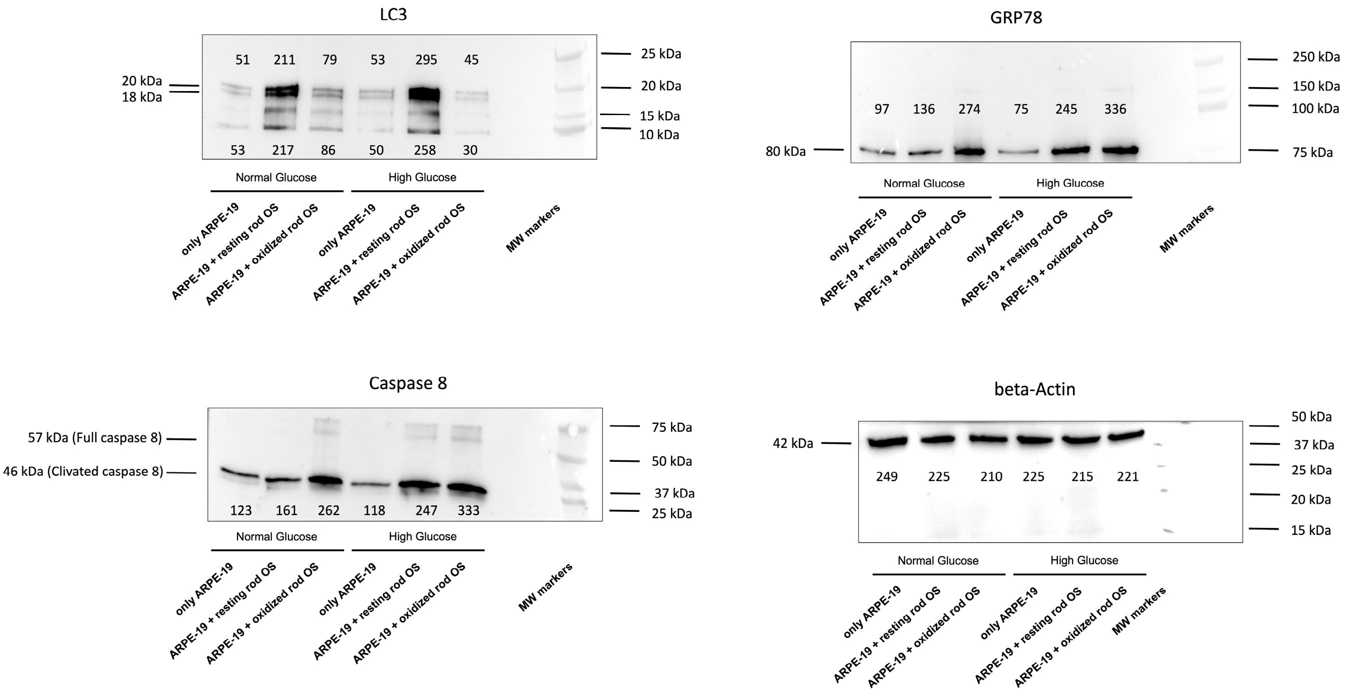

Figure S3. Whole WB signal against LC3, GRP78, and cleaved caspase 8 expression in ARPE-19 grown in normal- and high-glucose media and incubated with UOx- or Ox-rod OSs. WB signals refer to Panel A in Figure 6 of the manuscript.

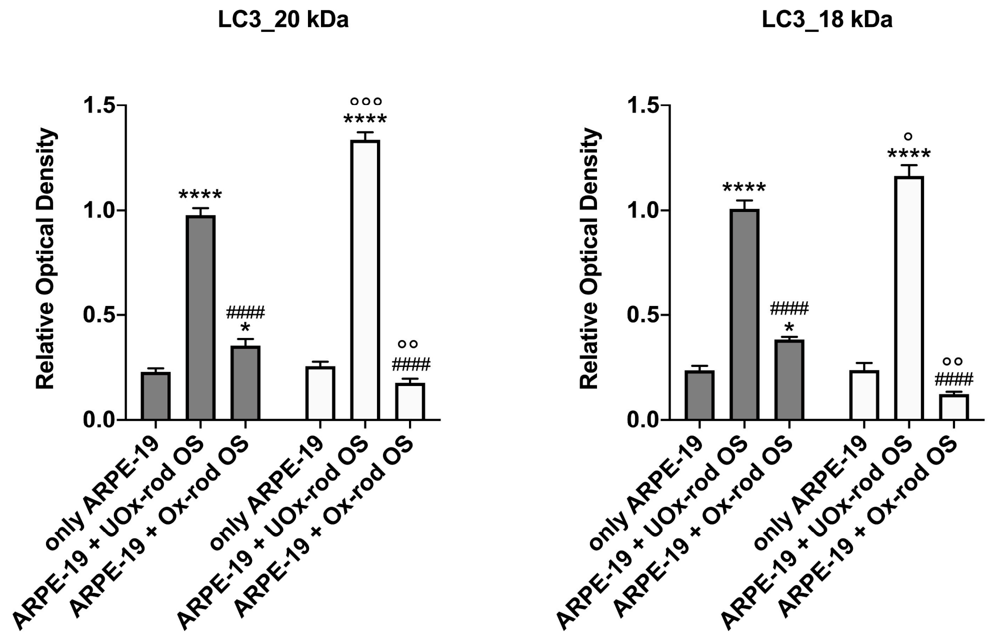

Figure S4. Densitometric analysis of 20 and 18 kDa bands of the WB analysis against LC3 in ARPE-19 grown in normal- and high-glucose media and incubated with UOx- or Ox-rod OSs. Densitometric analysis of 20 kDa and 18 kDa LC3 bands are reported in Figure 6. Gray and white columns represent ARPE-19 cells grown in NG or HG media, respectively. Data are expressed as the mean ± SD and are representative of four independent replicates (n = 4). * and **** indicate a $p < 0.05$ or 0.0001, respectively, between signals in ARPE-19 cells incubated or not with rod OSs; #### indicates a $p < 0.0001$ between the ARPE-19 cells incubated with UOx- or Ox-rod OSs; °, °°, and °°° indicate a $p <$ 0.05, 0.01, or 0.001, respectively, between the signal intensity in ARPE-19 cells grown in NG or HG media.
